# Climate change models predict decreases in the range of a microendemic freshwater fish in Honduras

**DOI:** 10.1038/s41598-020-69579-7

**Published:** 2020-07-29

**Authors:** Caleb D. McMahan, César E. Fuentes-Montejo, Luke Ginger, Juan Carlos Carrasco, Prosanta Chakrabarty, Wilfredo A. Matamoros

**Affiliations:** 1 Field Museum of Natural History, 1400 S. Lake Shore Dr., Chicago, IL USA; 2 Escuela de Biología, Facultad de Ciencias Químicas y Farmacia, Universidad de San Carlos de Guatemala, Edificio T10, Ciudad Universitaria, Zona 12, 01012 Ciudad de Guatemala, Guatemala; 3 Heal the Bay, 1444 9th Street, Santa Monica, CA USA; 4 Departamento de Biología, Facultad de Ciencias del Mary Ambientales, CASEM, Universidad de Cádiz, Puerto Real, 11510 Cádiz, Spain; 5 Instituto Técnologico Superior de Tela, Universidad Nacional Autónoma de Honduras, Boulevard Suyapa, Tegucigalpa, Honduras; 6 LSU Museum of Natural Science, Department of Biological Sciences, Louisiana State University, Baton Rouge, LA 70803 USA; 7 Instituto de Ciencias Biológicas, Universidad de Ciencias y Artes de Chiapas, Libramiento Norte Poniente 1150, Col. Lajas Maciel, C.P. 29039 Tuxtla Gutiérrez, Chiapas Mexico; 8 Maestría en Ciencias en Biodiversidad y Conservación de Ecosistemas Tropicales, Instituto de Ciencias Biológicas, UNICACH, Libramiento Norte # 1150, Col. Lajas Maciel, C.P. 29039 Tuxtla Gutiérrez, Chiapas México

**Keywords:** Ecological modelling, Tropical ecology, Biogeography

## Abstract

Despite their incredible diversity, relatively little work has been done to assess impacts of climate change on tropical freshwater organisms. *Chortiheros wesseli* is a species of Neotropical cichlid (Cichlidae: Cichlinae) restricted to only a few river drainages in the Caribbean-slope of Honduras. Little is known about this species and few specimens had been collected until recently; however, our work with this species in the wild has led to a better understanding of its ecology and habitat preferences making it an excellent model for how freshwater fishes can be affected by climate change. This study assesses the distribution and habitats of *Chortiheros wesseli* using a combination of field data and species distribution modeling. Results indicate this species is largely limited to its current range, with no realistic suitable habitat nearby. Empirical habitat data show that this species is limited to narrow and shallow flowing waters with rapids and boulders. This habitat type is highly influenced by precipitation, which contributed the greatest influence on the models of present and future habitat suitability. Although several localities are within boundaries of national protected areas, species distribution models all predict a reduction in the range of this freshwater fish based on climate change scenarios. The likelihood of a reduced range for this species will be intensified by adverse changes to its preferred habitats.

## Introduction

Current and projected impacts of climate change on fishes have been described in numerous studies and thoroughly synthesized by Myers et al.^1^. Those authors demonstrate that many of the studies on freshwater fishes (few relative to marine studies) have focused on species of economic concern (e.g. salmon) and have largely targeted North America and Europe^1^. This leaves vast geographic and taxonomic voids in our understanding of ongoing and future climate change impacts on freshwater biota, particularly in mega-diverse tropical systems^1^. Evidence suggests tropical organisms may be as, or more, vulnerable to climate change impacts as temperate biota^2,3^.

The distributions of organisms depend on a variety of interacting abiotic and biotic variables (e.g. evolution, physiography, climate, habitat, competition^4,5^) and provide key baseline data for documenting effects of climate change. However, combinations of those variables driving distributional patterns are far less well understood, particularly for aquatic taxa. Empirical studies (combining in situ examination of wild animals and modeling) assessing these variables are important sources of information for testing a range of hypotheses, as well as for formulating conservation priorities and strategies. Such empirical studies are not always possible given the time and resources necessary to gather such ecological data.

Species Distribution Models (SDMs) represent the probability of species occurrence across a spatial landscape based on responses to environmental variables. These SDMs can be a robust source of information in the absence of empirical ecological data^5–7^. In addition to contemporary distributions^8,9^, models can be projected onto paleo- and future landscapes to test hypotheses related to temporal range shifts based on changing conditions (e.g. glaciation cycles, future climate predictions^10,11^). For example, such distributional models predict a future decrease in suitable habitat based on estimated future shifts in climate for *Engraulis ringens*, an anchovy from Chilean waters^12^, as well as for *Karsenia koreana*, the only plethodontid salamander in Asia^13^. Constructing SDMs for aquatic taxa can be difficult because of a limited set of ambient variables and a terrestrial focus; however, with robust occurrence data these layers have offered novel insights into biogeographic patterns in fishes and amphibians^14–16^.

**Figure 1:**
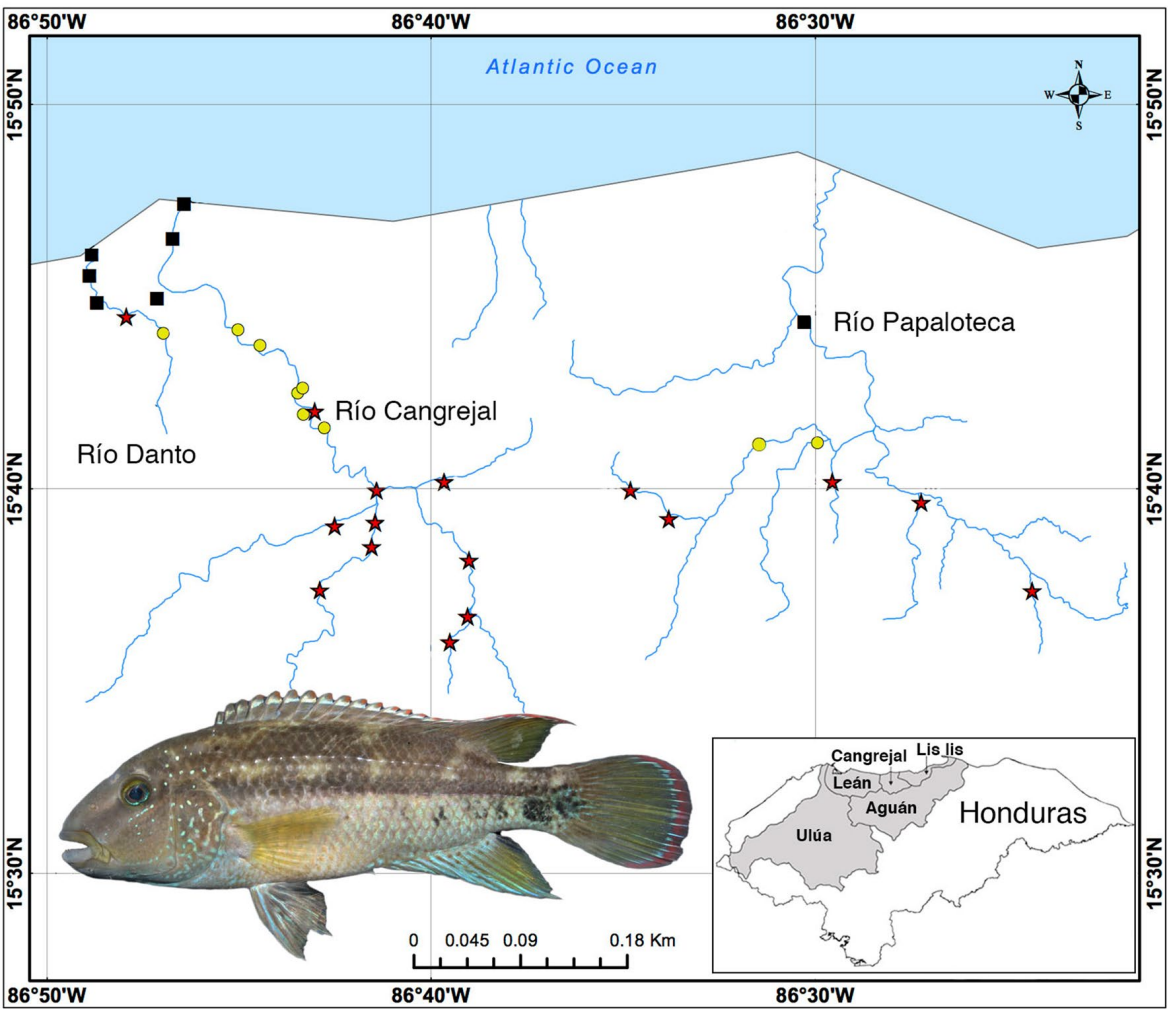
Map showing localities for *Chortiheros wesseli* samples and relevant river drainages in Honduras (generated in ArcMap 10.7). Black squares indicate absence, yellow circles indicate ‘less abundant’, and red stars indicate ‘abundant’ sample sizes of *C*. *wesseli*. Insert is a live individual of *C*. *wesseli* (LSUMZ 14519, 70.68 mm SL); photo courtesy of D. Smith.

In this study we focus on *Chortiheros wesseli*, a cichlid species known only from a small montane region in the Caribbean slope of Honduras (Fig. 1). Little is known about the biology of this microendemic fish beyond general observations in the original description^17^. All the original specimens came from the same general location, precluding comparison to additional localities. Future climate change predictions suggest drastic changes in patterns of rain and drought in Central America^18^, and given the narrow distribution of this endemic species in Honduras (one of the most limited of all Central American freshwater fishes), it is likely this species will be greatly affected by increasing variability in climatic and ecological conditions. Our aims were (1) to elucidate fine-scale patterns of habitat association for this species based on thorough fieldwork and environmental data, and (2) to integrate fieldwork and modeling to assess the current distribution of *C*. *wesseli* and test the potential impact of climate change on the range of this narrowly endemic species.

## Results

### Habitat and ecology

**Figure 2:**
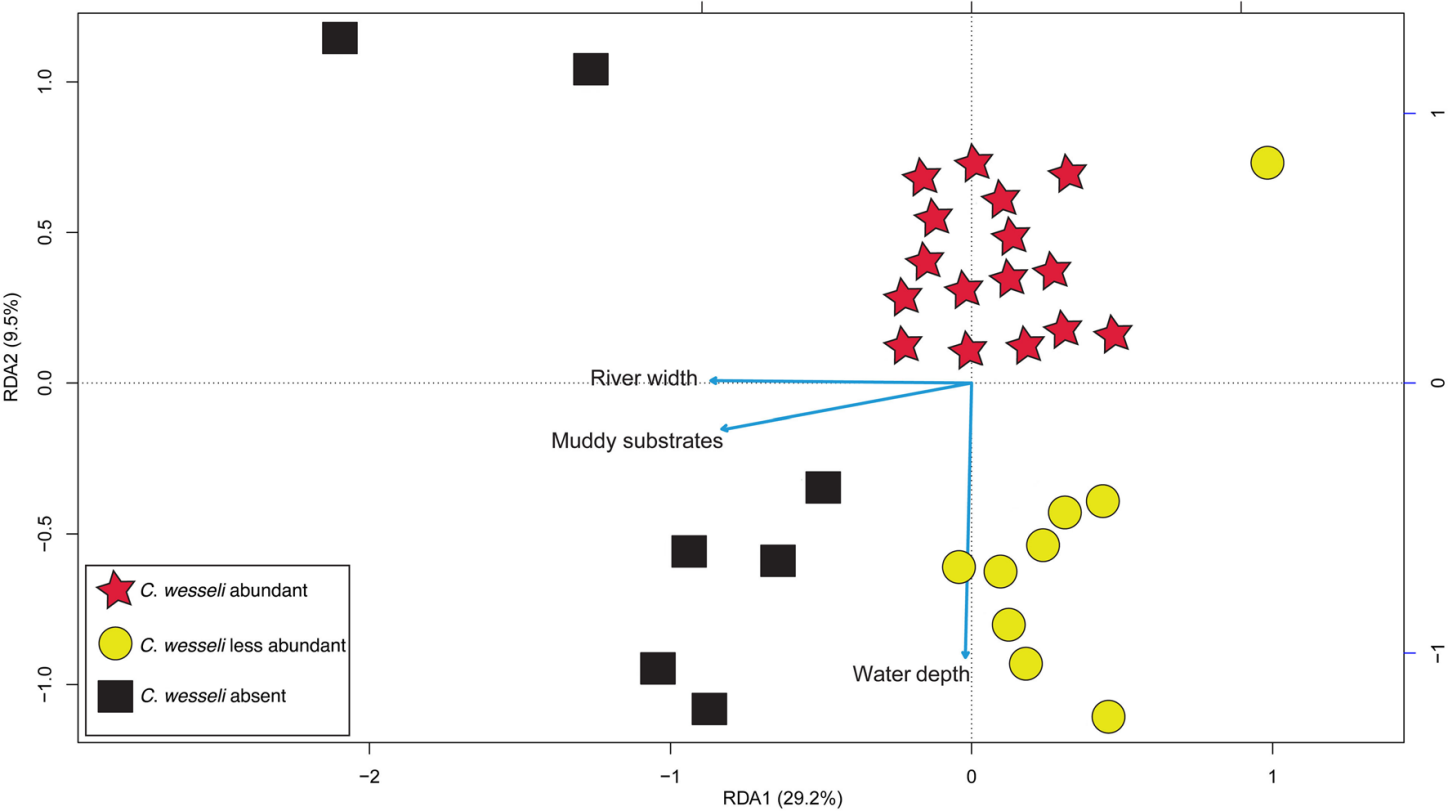
Triplot showing results of the redundancy analysis (RDA). Symbols are the same as those used in Fig. 1.

Field collections of *C*. *wesseli* at each locality were categorized as ‘less abundant’ (N = 1–10) and ‘abundant’ (N =  > 10) (Fig. 1). The parsimonious RDA (Fig. 2) explained 38.7% of variation, of which 29.2% was explained by axis 1 and 9.5% by axis 2. Constrained variance totaled 43.3% whereas the unconstrained variance was 56.6%. The forward selection procedure selected three environmental factors as contributing the most to the output model: river width, mud substrate, and water depth. Results of the RDA suggested that *C*. *wesseli* was limited to relatively shallow localities where the river width was narrow and the dominant substrate consisted of rocks and boulders. These localities were consistent with middle and upper reaches of the rivers.

### Models of current and future habitat suitability

**Figure 3:**
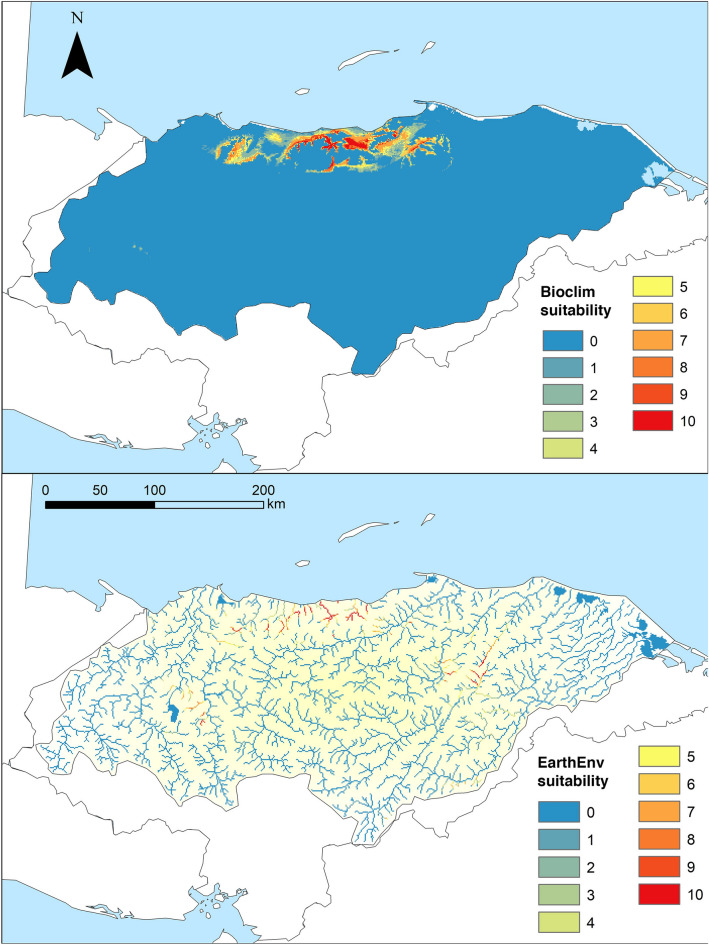
Maps showing suitable habitat for *Chortiheros wesseli*, under Bioclim layers (upper) and EarthEnv layers (lower). Maps generated in ArcMap 10.7.

Suitable environments for *C*. *wesseli* based on SDMs showed a narrow distribution using Bioclim variables, with the Cangrejal and Lis Lis river basins as the most suitable environments, followed by the Leán and Aguán river basins (Fig. 3). Contemporary models with EarthEnv variables suggested similar results but with less suitable habitat throughout the Aguán River. Both models indicated suitable environments in distant areas such as the Ulúa River but in all cases with lower suitability at the edges.

**Figure 4:**
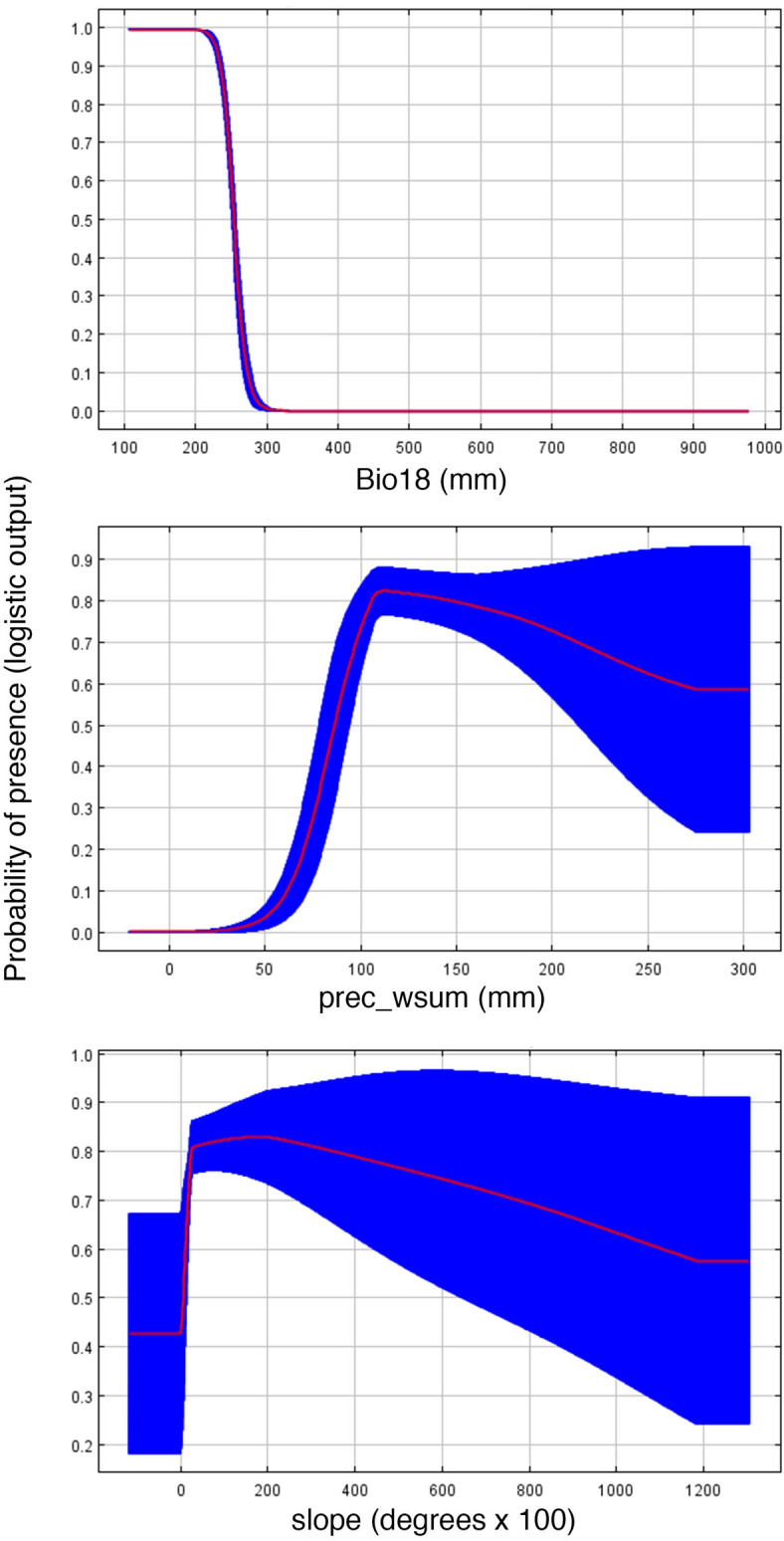
Response curves of three influential environmental predictors used for species distribution models for *Chortiheros wesseli*. Abbreviations defined in Supplementary Table S2.

Contemporary models using Bioclim variables recovered a total of 3,686.81 km^2^ of suitable habitat across the study area (Table 1), with an AUC of 0.997 ± 0.001 and a TSS of 0.936 ± 0.071. In contrast, models based on EarthEnv variables recovered only 1,276.2 km^2^ of suitable habitat across the study area (Table 1), with an AUC of 0.988 ± 0.004 and a TSS of 0.751 ± 0.159. Results of jackknife analysis showed the Bioclim variable with the highest contribution to the model was Bio18 (precipitation during the warmest quarter; 49.1%), followed by Bio04, Bio15, Bio19, and Bio09, all with < 10% contribution (related to rainfall and temperature seasonality). Bio18 was additionally the most important based on average permutation (Fig. 4). Remaining variables contributed < 5% to the model. The EarthEnv variable with greatest contribution to the model was prec_wsum (related to rainfall; 35.4%), followed by Slope, LC_wavg (landcover), and Tmin_wavg (temperature), all with < 20% contribution (Fig. 4). The remaining variables had either a low (< 10%; prec_sum, LC_ran), very low (< 4%), or no contribution (Soil_avg, Soil_max, Hydavg, LC_min, LC_max; related to soil content, landcover, and hydrology). Based on EarthEnv variables, prec_wsum had the highest average permutation importance (63%), followed by Tmin_wavg (11.4%) and Soil_ran (11.1%).

**Table 1:** Suitable area in km^2^ and percentage of covered area with respect to the present-day suitable area.

Suitability	Present	RCP2.6				RCP8.5			
		2050	%	2070	%	2050	%	2070	%
**Suitability over the complete study area**									
Low	4,175.14	1,674.75	40.11	2,369.49	56.75	3,512.29	84.12	1,504.43	36.03
Mid	1,128.53	687.19	60.89	247.52	21.93	1,219.15	108.03	317.16	28.1
High	1,505.27	436.31	28.98	689.7	45.82	1,027.84	68.28	571.4	37.96
Total	6,808.94	2,798.25	41.1	3,306.72	48.56	5,759.28	84.58	2,392.99	35.14
**Suitability inside of protected areas**									
Low	811.37	205.57	25.34	249.2	30.71	376.74	46.43	172.01	21.2
Mid	172.01	158.58	92.19	57.89	33.66	119.98	69.75	58.73	34.14
High	252.56	151.87	60.13	246.68	97.67	311.29	123.26	223.19	88.37
Total	1,235.93	516.02	41.75	553.78	44.81	808.01	65.38	453.93	36.73

Suitability	Present (total area)	Present (protected areas)	%						
**Suitability described by Earth Environment models**									
Low	1,606.79	57.06	3.55						
Mid	745.92	53.7	7.2						
High	399.39	74.68	18.7						
Total	2,752.1	185.43	6.74

Two national protected areas encompassing suitable habitat, Parque Nacional Nombre de Dios and Parque Nacional Pico Bonito, were recovered by our SDMs. The total coverage of suitable habitats inside of protected areas based on Bioclim and EarthEnv variables was 610.83 km^2^ and 111.59 km^2^, respectively (Table 1). Of the 25 occurrence records (locality points) used for building our SDMs, eight (32%) were located inside protected areas.

**Figure 5:**
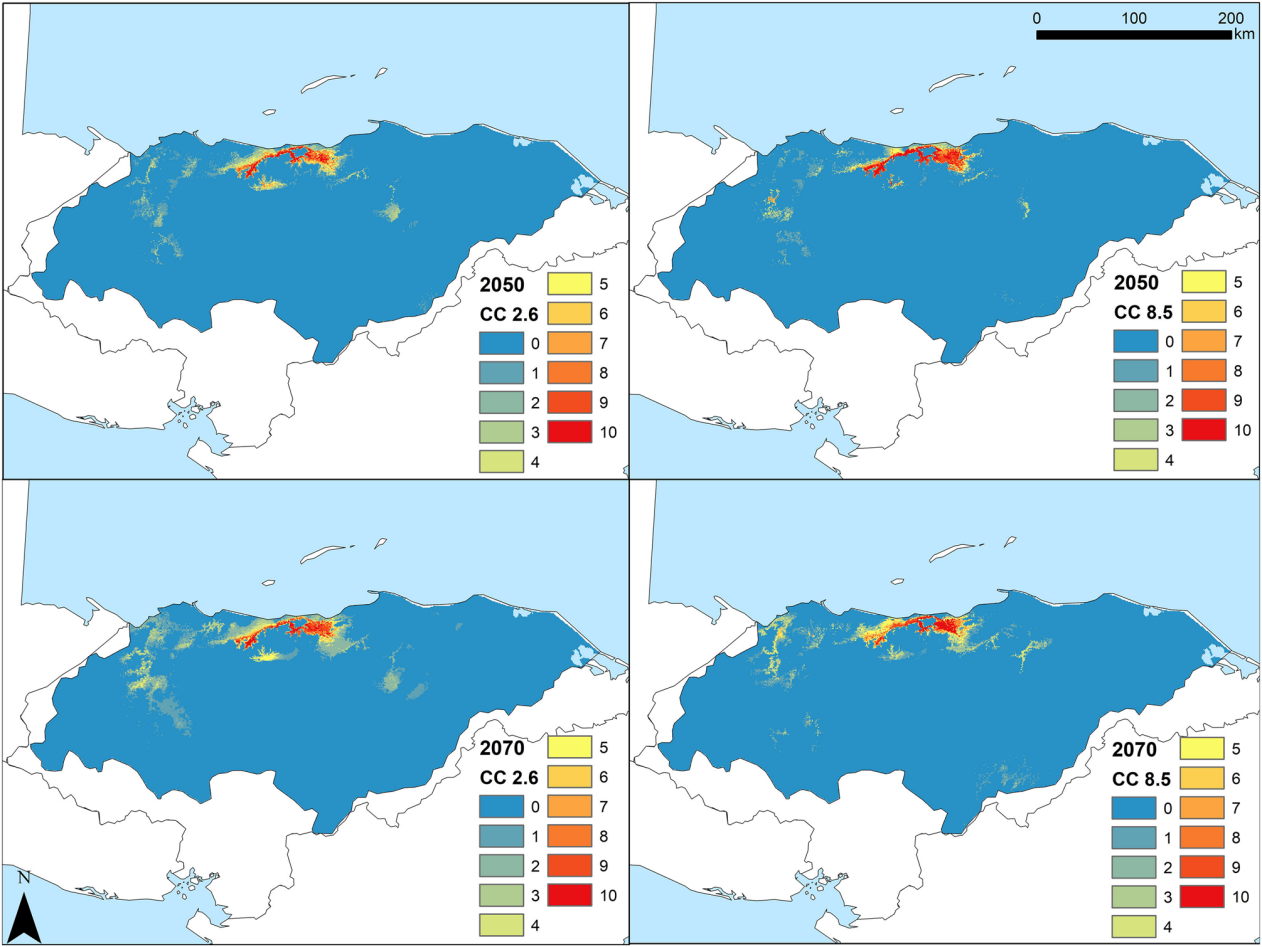
Maps showing suitable habitat for *Chortiheros wesseli* under future scenarios of climate change. Projections of suitable environment for 2050 (top) and 2070 (bottom), for both RCP2.6 (left) and RCP8.5 (right). Maps generated in ArcMap 10.7.

**Figure 6:**
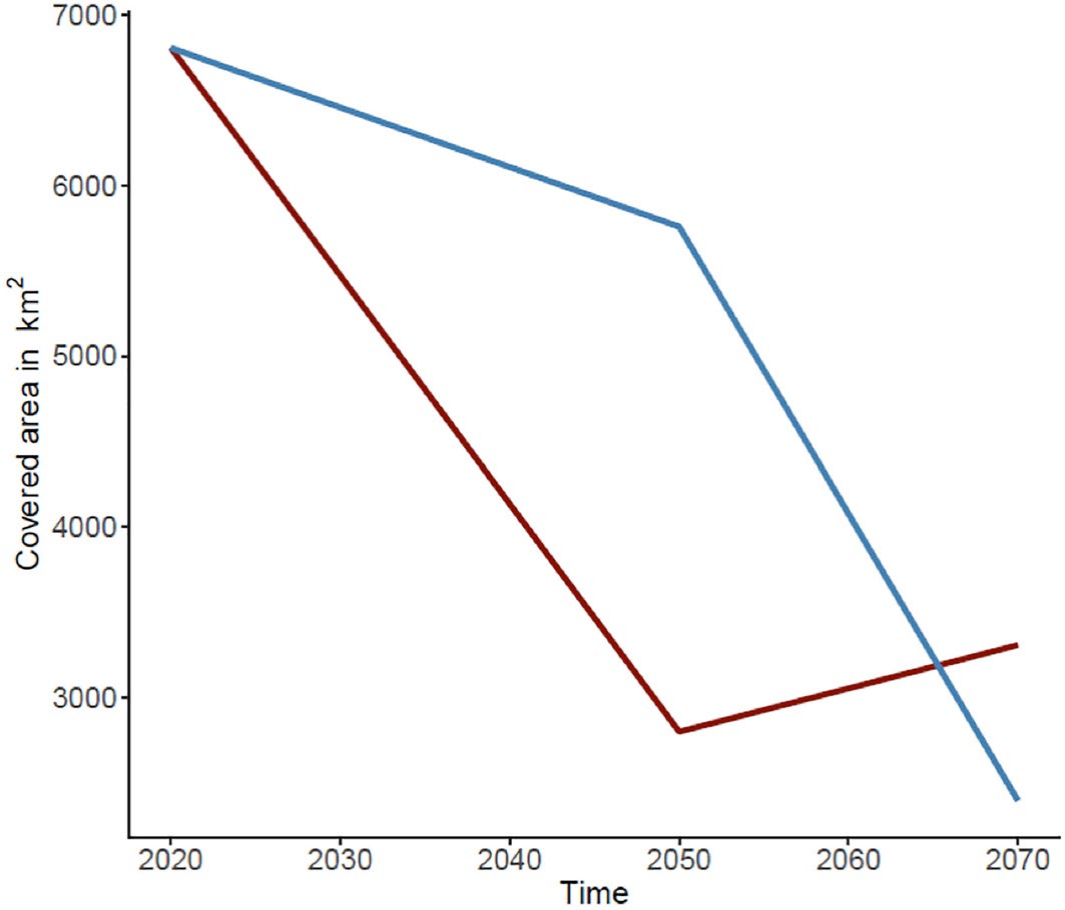
Comparison of total covered area of suitable habitats predicted by SDMs under RCP2.6 (red line) and RCP8.5 (blue line) scenarios.

Future SDMs predicted a reduction of environmental suitability, shifting from a wider area in the present to a substantially narrower area of suitable environment for *C. wesseli* by 2050 and 2070 (Fig. 5). Models based on RCP2.6 showed a severe decline in the range of suitable habitat by 2050 and a small increase by 2070, while models based on RCP8.5 showed a slight decrease in suitable habitat by the year 2050 and an abrupt, drastic reduction by the year 2070, leading to the narrowest area of suitable habitat predicted (Figs. 5, 6; Table 1). Overall both models converged in their predictions of decreases in the range of *C*. *wesseli* (Fig. 6).

## Discussion

Freshwater ecosystems are among the most threatened in the world^19,20^. The Neotropics are known as an incredible hotspot for biodiversity, particularly in the case of an extraordinary species richness for fishes in Central America^21,22^; however, given the short ranges of species' habitats and continuous threats (e.g. habitat loss, pollution, overexploitation), this area is considered one of great concern for its freshwater biodiversity^23^. This concern is deepened based on climate change predictions that will produce unfavorable futures for several taxa due to issues such as loss of habitat and transformed food web dynamics^24,25^.

Our results illuminate habitat characterizations of a narrowly endemic Neotropical freshwater fish from the Caribbean slope of Honduras. Areas with the highest probability of occurrence are supported by our field observations, demonstrating the robustness of our model. The SDMs highlight a slightly larger range of suitable habitat than the currently known distribution of the species, but SDMs estimate the fundamental niche of a species, which likely differs from the realized niche^16,26^. The suitable habitat detected in rivers slightly west of the known distribution of *C*. *wesseli* can be attributed to shared environmental and physiographic characteristics^21^. However, extensive sampling in these rivers supports the absence of this species from those systems, and can be easily explained in aquatic taxa based on dispersal capabilities and vicariance^22,27^. The predicted range of this species could further be restricted by abiotic variables not included in our SDMs (e.g. water chemistry), as well as by biotic factors (e.g. species interactions, competition).

Most studies utilizing SDMs lack empirical habitat data that correspond to occurrence records, but with these data we can better assess suitable habitat within predicted (modeled) areas. We have documented habitat associations for this endemic cichlid that is found exclusively in aquatic habitats with rapids and riffles, as well as large rocks and boulders for shelter. This observation is consistent with the most important variables contributing to our SDMs. Given that both Bio18 and prec_wsum both represent variables related to rainfall (Fig. 4), we attribute this to the influence of precipitation on water depths and flow. Our results suggest that places with muddy bottoms and wide, deep river channels are not used by *C*. *wesseli* (Fig. 2). Based on our results we predict that the habitats for this species will be characterized by having a narrower channel width and shallower depth with a substrate dominated by large boulders and rocks. This kind of habitat is potentially threatened by climate change projections, especially in Central America, where seasonal variation is predicted to be considerably different in the future^18^. These conclusions are additionally supported by our results showing how changes in slope impact habitat suitability (Fig. 4). Small changes in elevation (mouths of rivers) provide only moderately suitable habitats for the species, but probability of occurrence increases as slope increases. However, probability of occurrence begins decreasing as slope continues increases (Fig. 4). These models are consistent with our fieldwork and habitat data (Fig. 1). Species are generally predicted to move to higher elevations as a response to changing climate^28,29^, but the presence of waterfalls and other barriers to moving upslope in the current habitat of this species will reduce its ability to disperse. Other species that are restricted to these habitats (e.g. bivalves, crayfish, aquatic beetles) presumably would be similarly endangered by future climate change.

The rivers encompassing the distribution of *C*. *wesseli* are part of the Motagua-Nombre de Dios Area of Endemism^21^ and two national parks (Pico Bonito and Nombre de Dios). However, the predicted patterns of reduction in habitat suitability and range will likely be felt inside protected areas, as well. Ongoing conservation efforts in this area should be enhanced and promoted by the results of our study. While these environmental and habitat data indicate higher commonality and abundance of *C*. *wesseli* than previously known^30^, the localized specificity of habitat in this region of Honduras certainly supports maintaining conservation efforts for the area. Future SDMs indicate that this area will suffer effects from climate change that would reduce the suitable environment and range area for *C. wesseli*^24^, which highlights the importance of conservation efforts in the region that should include better managed water usage and waste management.

Our results explain the potential impacts of changing climate on the future distribution of *C. wesseli*; however, these models can say nothing regarding habitat use. Our environmental field data clearly demonstrate habitat specificity for this endemic cichlid within the rivers in which it occurs. One of the most concerning aspects of climate change in Central America is the likelihood of changes in seasonal dynamics of rainy and dry seasons. These changes are more likely to modify the availability and quality of habitats in the actual ranges of *C. wesseli*, shrinking the distribution of the species and potentially reducing the size of populations^31, 32^, which should be of importance throughout ongoing and future conservation planning.

This study demonstrates that high quality distributional data that can be used for garnering a better ecological understanding of a species can also be used to predict future effects of climate change. The species studied here is an excellent example of a narrowly endemic freshwater species that, despite a limited distribution, can have big impacts on our understanding of a much larger region well into an uncertain future.

## Methods

### Fieldwork survey

In September 2011 we sampled 32 localities; four in the Río Danto, 20 in the Río Cangrejal (both part of the Cangrejal river basin) and eight in the Río Papaloteca (Lis Lis river basin; Fig. 1). Sampling was carried out with a combination of collecting gear: electro-fisher, seine, castnet and spear. In each locality we sampled a stretch of river approximately 150 m in length. In order to homogenize sampling efforts, we performed 20 castnet throws, one pass with the seine and one pass with the electro-fisher. Additionally, one individual spear-fished for *C. wesseli* for up to 30 min. Since our goal was to characterize the habitat where *C. wesseli* was found, we sampled localities where we expected the fish to be present but also included localities with different environments where we hypothesized the fish to be absent (e.g. near river mouths). Specimens were deposited in the Louisiana State University Museum of Natural Science (LSUMZ). All methods were carried out in accordance with relevant guidelines and regulations in Honduras, and all protocols were approved and conducted under Institutional Animal Care and Use Committee (IACUC) approval 09-022 at Louisiana State University.

### Environmental data and analysis

To ensure adequate characterization of habitats in which *C. wesseli* was present, we collected an array of environmental data at each locality: river width, water depth, estimation of canopy cover, erosion, estimation of habitat availability, dominant substrates (percentage of bed rock, boulders, rocks, cobble, gravel, sand, mud and silt), and river condition (run, waters falls, rapids, riffles and pools). To estimate slope, we measured the altitude in meters above sea level at the sampling point and an additional two points 500 m above and below the sampling point. Slope was represented as a percentage [slope = (U − D/1,000) × 100], where U = altitude 500 m upstream of sampling point and D = altitude 500 m downstream of sampling point.

We implemented a Redundancy Analysis (RDA^33^) to assess the relationship between environmental factors and the presence of *C. wesseli* in a given locality. This procedure uses a matrix with all abundance values (independent variables) captured in our samples, and all recorded environmental data (dependent variables) as explained above. Since environmental data are often correlated, we implemented a parsimonious RDA^34^ to eliminate highly correlated factors and obtain a model in which a reduced, less correlated set of environmental factors explained the same amount of variance as when using all environmental factors. In order to detect the best-fit model we applied a forward selection procedure within the global RDA in R^35^ using the ‘ordistep’ function in the package Vegan^36^. A new RDA was then performed with only those environmental factors chosen by the forward selection procedure.

### Current and projected habitat suitability

We used species distribution models (SDMs) as an independent assessment of current suitable environments for *C*. *wesseli* in Honduras, as well as to test for shifts in the distribution of this narrowly-distributed endemic freshwater fish associated with future projections of changes in climate. Latitude and longitude for all specimens were compiled and all georeferenced points were examined for accuracy. A total of 25 occurrence points were used after removing duplicate points within the same grid cell (Supplementary Table S1). Species distribution models were estimated using Maxent v.3.4.1^37^, which overlays presence data onto environmental layers and characterizes those conditions most suitable for a species. We used 19 freshwater-specific environmental variables (Earth Environment (EarthEnv)^38^) and 19 bioclimatic variables (Bioclim^39^) to estimate contemporary suitable habitat (Supplementary Table S2). All layers were clipped to the extent of Caribbean basins in Honduras, from the Chamelecón to Patuca River basins, with a 30 arc second (~ 1 km) spatial resolution. This reduced the potential for pseudoabsences detected in analyses^14,16^. We tested for correlation among variables using a Pearson’s correlation test, and when two layers were correlated (threshold 0.8) we retained the climate layer that appeared most biologically meaningful and excluded layers with multiple correlations. The remaining variables (eight EarthEnv, six Bioclim) were used to estimate contemporary suitable habitat (Supplementary Fig. S1). Comparisons of the full dataset and reduced dataset demonstrated clear overestimation of distribution and suitable habitat in the reduced dataset of fewer variables; highly supported by our empirical field and habitat data. This is in contrast to some views that correlated variables can overestimate ranges; however, recent studies support the robustness of Maxent in optimizing collinearity among variables, thus removing highly correlated variables from the complete dataset has little impact^40,41^. Therefore, we used the full dataset for analyses as this most accurately matched biology and distribution of the species, particularly working at such a fine scale, thus allowing Maxent to choose the most informative variables among all predictors for modeling distribution^15^. We ran Maxent with a convergence threshold of 10^−6^ and used 10,000 iterations with bootstrap resampling and 10 replicates^42,43^, with 25 independent presence records for each replicate to avoid duplicated records and used a 30% random test percentage of these records to assess model performance.

Probable future distribution was estimated using two different climate change scenarios known as Representative Concentration Pathways (RCPs), which consider the impacts of climate change strategies on greenhouse gas emissions (GGE) and published by the UN's Intergovernmental Panel on Climate Change. We used the corresponding bioclimatic layers from the Community Climate System Model 4.0 (CCSM4), a coupled General Circular Model (GCM) that uses four sub-models (atmosphere, land, ocean, sea-ice) to simulate climatic conditions, under the Coupled Model Intercomparison Project Phase 5 (CMIP5) downscaling. In order to compare two different scenarios with contrasting climate change patterns, we used RCP2.6 (the minimum GGE scenario; less drastic climatic variation) and RCP8.5 (the maximum GGE scenario; unchecked climatic variation) for the years 2050 (average 2040–2060) and 2070 (average 2060–2080)^24^.

We used the receiver-operating characteristic curve (AUC) as an evaluation of model performance^44^. The AUC values were between 0 and 1, with higher values indicating a better model performance. When the AUC was below 0.5, the model performed worse than random, and the closer the AUC was to 1 the better the model performed^45^. We additionally used the True Skill Statistic (TSS) as an independent assessment of model performance, with values ranging between − 1 and + 1, where + 1 indicated perfect agreement and values ≤ 0 indicated a performance no better than random^46^. We then reclassified each replicate into binary (presence/absence) maps to evaluate suitability using the Maximum Training Sensitivity Plus Specificity threshold, which minimizes false-presence and false-absence errors^16,47,48^, and stacked them to produce a single map. We classified suitable area into five categories as low (1–2 overlapping replicates), low-mid (3–4), mid (5–6), mid-high (7–8), and high (9–10), with the sum equaling total suitable area predicted. We clipped the covered area that fell inside the main protected areas distributed across the Honduran Caribbean coast near collecting localities, and also estimated the suitable area inside these protected areas. We then compared the number of presence records inside protected areas with records outside of them. Finally, we estimated the covered area of suitable habitat for the present-day Bioclim model and the two RCP scenario models, comparing the total coverage area of future models with the present area covered.

## Supplementary information

Supplementary Information.
