# Supplementary material for: Climate change models predict decreases in the range of a microendemic freshwater fish in Honduras
Source: Sci Rep. 2020 Jul 29;10:12693. doi: 10.1038/s41598-020-69579-7 (PMC7391645; doi:10.1038/s41598-020-69579-7)

**Climate change models predict decreases in the range of a microendemic freshwater fish in Honduras**

Caleb D. McMahan^1^*, César Fuentes Montejo^2^, Luke Ginger^3^, Juan Carlos Carrasco^4,5^, Prosanta Chakrabarty^6^, and Wilfredo A. Matamoros^7,8^

^1^*Field Museum of Natural History, 1400 S. Lake Shore Dr., Chicago IL USA. cmcmahan@fieldmuseum.org.*

^2^*Escuela de Biología, Facultad de Ciencias Químicas y Farmacia, Universidad de San Carlos de Guatemala, Edificio T10, Ciudad Universitaria, zona 12, 01012 Ciudad de Guatemala, Guatemala.*

^3^*Heal the Bay, 1444 9th Street. Santa Monica CA USA.*

^4^*Departamento de Biología, Facultad de Ciencias del Mar y Ambientales, CASEM, Universidad de Cádiz. Puerto Real, 11510 Cádiz, España.*

^5^*Instituto Técnologico Superior de Tela, Universidad Nacional Autónoma de Honduras, Boulevard Suyapa, Tegucigalpa, Honduras*

^6^*LSU Museum of Natural Science, Department of Biological Sciences, Louisiana State University, Baton Rouge LA 70803 USA*

^7^ *Instituto de Ciencias Biológicas,* *Universidad de Ciencias y Artes de Chiapas, Libramiento Norte Poniente 1150, Col. Lajas Maciel, C.P. 29039, Tuxtla Gutiérrez, Chiapas, Mexico*

^8^*Maestría en Ciencias en Biodiversidad y Conservación de Ecosistemas Tropicales. Instituto de Ciencias Biológicas, UNICACH. Libramiento Norte # 1150. Col. Lajas Maciel. C.P. 29039, Tuxtla Gutiérrez, Chiapas, México.*

**Supplementary Table 1.** Museum catalog numbers and GPS coordinates for *Chortiheros* *wesseli* used in SDM analyses. LSUMZ, Louisiana State University Museum of Natural Science; UMMZ, University of Michigan Museum of Zoology; USM, University of Southern Mississippi.

| Catalog No. | Latitude | Longitude |
| --- | --- | --- |
| LSUMZ 15641 | 15.655306 | -86.563778 |
| LSUMZ 15646 | 15.671778 | -86.655889 |
| LSUMZ 15653 | 15.660778 | -86.4539712 |
| LSUMZ 15672 | 15.692222 | -86.504972 |
| LSUMZ 15674 | 15.652139 | -86.707444 |
| LSUMZ 15681 | 15.742139 | -86.792361 |
| LSUMZ 15702 | 15.641639 | -86.692306 |
| LSUMZ 15733 | 15.693056 | -86.712972 |
| LSUMZ 15710 | 15.622861 | -86.714833 |
| LSUMZ 15716 | 15.665972 | -86.580417 |
| LSUMZ 15869 | 15.686583 | -86.499111 |
| UMMZ 223306 | 15.686505 | -86.686505 |
| UMMZ 234760 | 15.738898 | -86.510867 |
| USM 45584 | 15.73499 | -86.78217 |
| USM 35514 | 15.72891 | -86.74105 |
| USM 31561 | 15.70839251 | -86.7247207 |
| USM 31017 | 15.69885134 | -86.72205505 |
| USM 31022 | 15.70039066 | -86.71690134 |
| USM 35528 | 15.666091 | -86.690275 |
| USM 31574 | 15.65198798 | -86.69060953 |
| USM 31009 | 15.66977207 | -86.66093745 |
| USM 31003 | 15.63572643 | -86.65014314 |
| USM 35538 | 15.6124 | -86.65021 |
| USM 45575 | 15.60542 | -86.64486 |
| USM 34013 | 15.68609 | -86.52441 |

**Supplementary Table 2.** Environmental layers used to build species distribution models (SDMs) for *Chortiheros wesseli* in Caribbean river basins of Honduras.

|  | **Bioclim** | | **Earth Environment** | |
| --- | --- | --- | --- | --- |
| **No.** | **Short** | **Full** | **Short** | **Full** |
| 1 | Bio01 | Annual Mean Temperature | Hydavg | Hydroclimatic variables (average and sum) |
| 2 | Bio02 | Mean Diurnal Range (Mean of monthly (max temp - min temp)) | Hydwavg | Hydroclimatic variables (distance-weighted average and sum) |
| 3 | Bio03 | Isothermality (Bio2/Bio7)(*100) | LC_avg | Upstream landcover coverage (average) |
| 4 | Bio04 | Temperature Seasonality (standard deviation *100) | LC_max | Upstream landcover coverage (maximum) |
| 5 | Bio05 | Max Temperature of Warmest Month | LC_min | Upstream landcover coverage (minimum) |
| 6 | Bio06 | Min Temperature of Coldest Month | LC_ran | Upstream landcover coverage (range) |
| 7 | Bio07 | Temperature Annual Range (Bio5-Bio6) | LC_wavg | Upstream landcover coverage (distance-weighted average) |
| 8 | Bio08 | Mean Temperature of Wettest Quarter | Pre_sum | Monthly upstream precipitation (sum) |
| 9 | Bio09 | Mean Temperature of Driest Quarter | Pre_wsum | Monthly upstream precipitation (distance-weighted sum) |
| 10 | Bio10 | Mean Temperature of Warmest Quarter | Slope | Stream length and flow accumulation |
| 11 | Bio11 | Mean Temperature of Coldest Quarter | Soil_avg | Upstream soil (average) |
| 12 | Bio12 | Annual Precipitation | Soil_wavg | Upstream soil (distance-weighted average) |
| 13 | Bio13 | Precipitation of Wettest Month | Soil_max | Upstream soil (maximum) |
| 14 | Bio14 | Precipitation of Driest Month | Soil_min | Upstream soil (minimum) |
| 15 | Bio15 | Precipitation Seasonality (Coefficient of Validation) | Soil_ran | Upstream soil (range) |
| 16 | Bio16 | Precipitation of Wettest Quarter | Tmax_avg | Monthly maximum temperature (average) |
| 17 | Bio17 | Precipitation of Driest Quarter | Tmax_wavg | Monthly maximum temperature (distance-weighted average) |
| 18 | Bio18 | Precipitation of Warmest Quarter | Tmin_avg | Monthly minimum temperature (average) |
| 19 | Bio19 | Precipitation of Coldest Quarter | Tmin_wavg | Monthly minimum temperature (distance-weighted average) |

**Supplementary Figure 1.** Results of SDMs (present-day above and future below) with a reduced dataset after removing correlated variables. These results show an overestimate of distribution and suitable habitat, as evidenced by the field and habitat data for *Chortiheros* *wesseli* presented in this study. Maps generated in ArcMap 10.7.


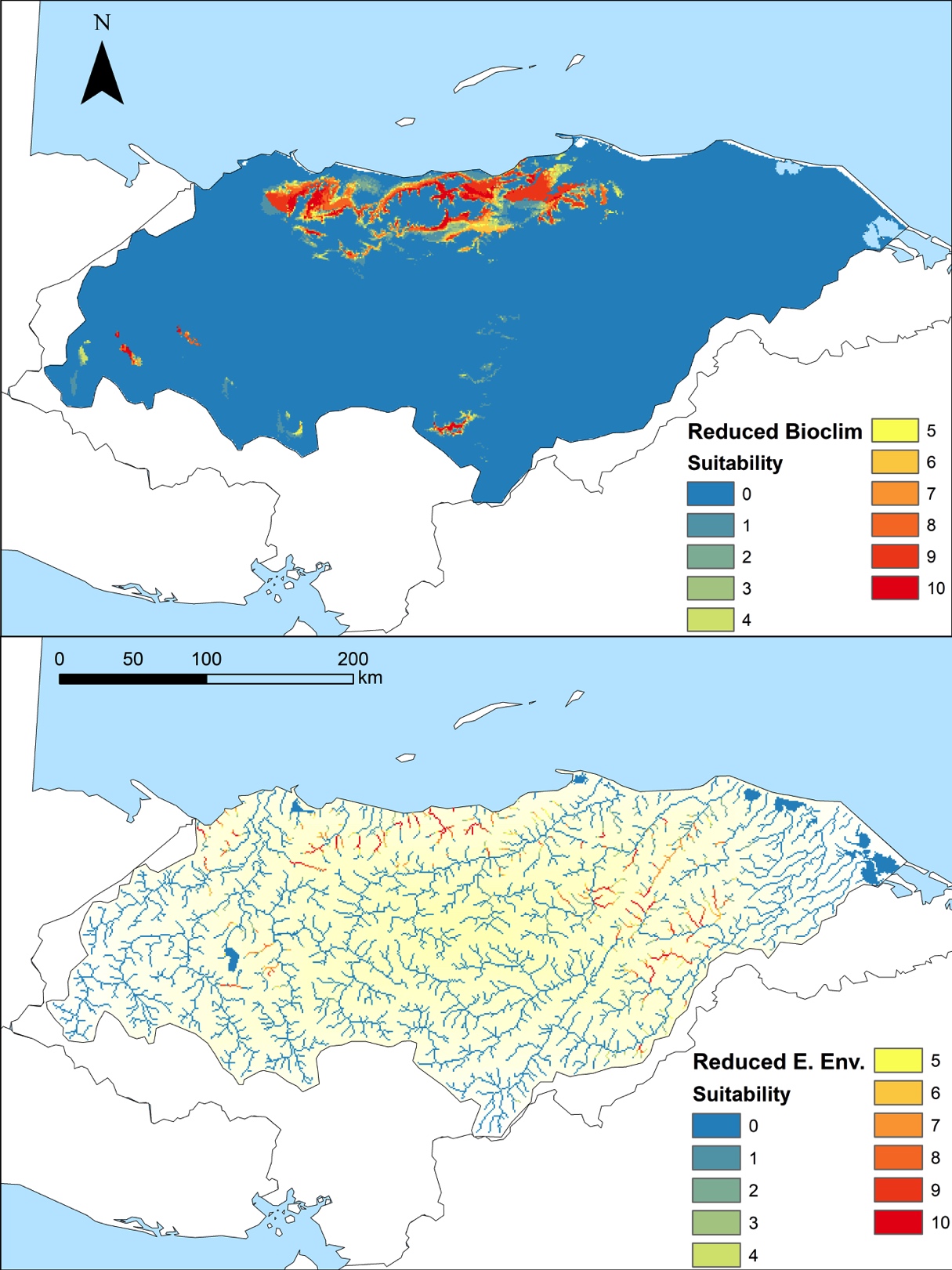


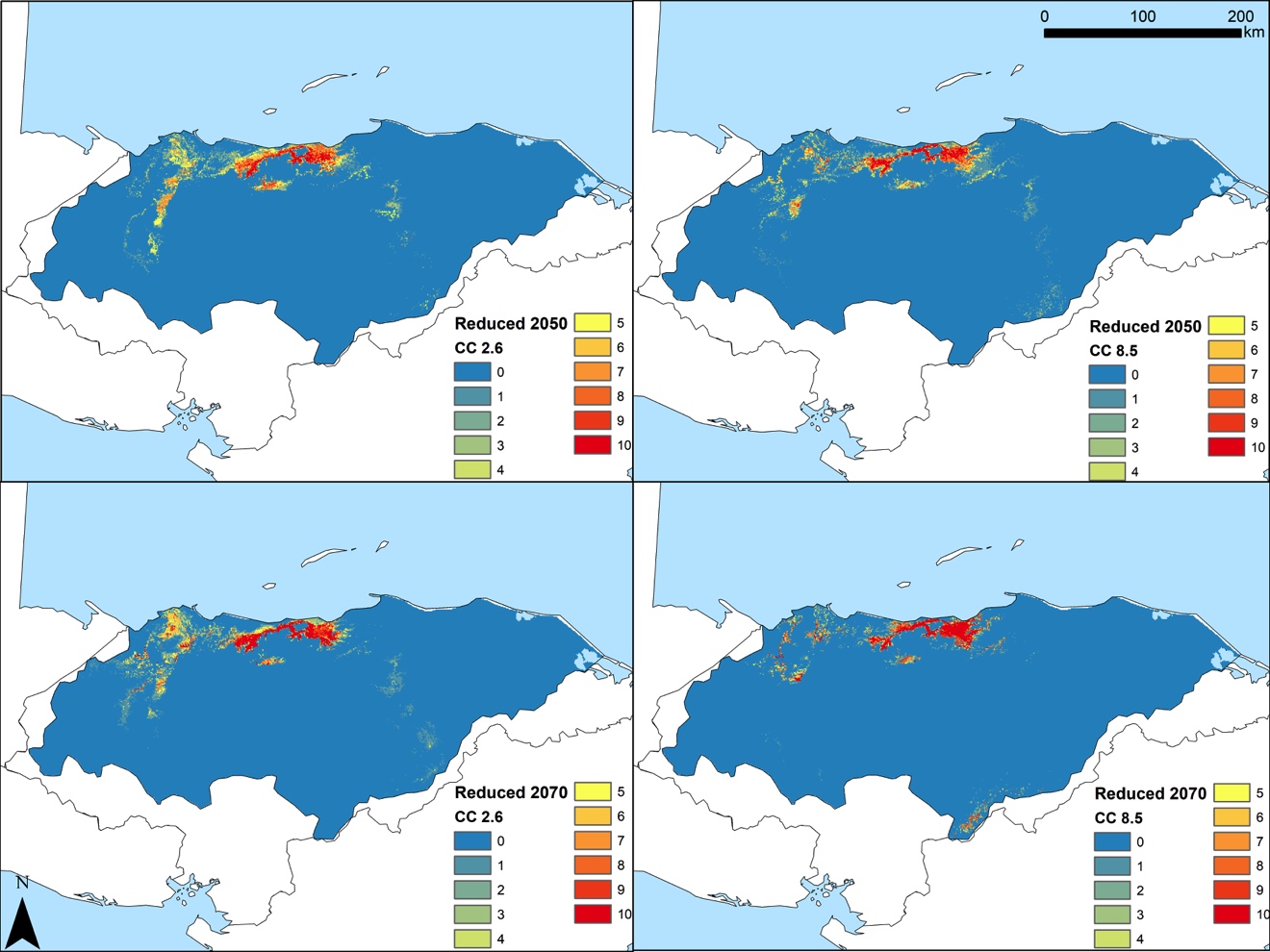

Supplement: Supplementary file 1 — Supplementary Information. [file 41598_2020_69579_MOESM1_ESM.docx]
